# ﻿Diversity and larval leaf-mining habits of Japanese jewel beetles of the tribe Tracheini (Coleoptera, Buprestidae)

**DOI:** 10.3897/zookeys.1156.97768

**Published:** 2023-03-29

**Authors:** Makoto Kato, Atsushi Kawakita

**Affiliations:** 1 Graduate School of Human and Environmental Studies, Kyoto University, Sakyo 606-8501, Kyoto, Japan Kyoto University Kyoto Japan; 2 The Botanical Gardens, Graduate School of Science, The University of Tokyo, Tokyo, 112-0001, Japan The University of Tokyo Tokyo Japan

**Keywords:** Agrilinae, *Habroloma*, leaf miner, mining pattern, *Symplocos*, *Trachys*

## Abstract

From the Japanese Archipelago, 12 *Habroloma* and 20 *Trachys* species (Buprestidae: Tracheini) have been recorded. Two new *Habroloma* species were found, which are associated with Elaeocarpaceae and Loranthaceae, also new host plant families/orders for Tracheini. The two new species are described as *Habrolomaelaeocarpusi***sp. nov.** and *Habrolomataxillusi***sp. nov.**, and the latter is the first Tracheini species shown to be associated with epiphytes. Leaf mines of 31 Tracheini species are also reported in this work, including new records of leaf mines for 16 Tracheini species. The larvae of all these recorded species are full-depth linear-blotch mesophyll miners of mature leaves and pupate within their mines. The mining habits of *Habroloma* species associated with *Symplocos* (Symplocaceae) are unique: the young larvae bore into midribs and petioles and cause leaf fall, and the larvae then mine the fallen leaves.

## ﻿Introduction

The coleopteran family Buprestidae is a species-rich clade whose larvae are xylophagous wood-borers, while the leaf-mining habit has evolved (Hering 1951). The tribe Tracheini of the subfamily Agrilinae is one of these leaf-mining clades and has great diversity, especially in Asia, Europe, and Africa. The two genera of Tracheini, *Trachys* and *Habroloma*, comprise more than 650 and 300 species worldwide, respectively (Bellamy 2008). Continental Asia is home to diverse trachyine species (Obenberger 1918, 1929) and the number of trachyine species has been underestimated. In recent years, 14 *Trachys* and 33 *Habroloma* species have been newly described from China (Peng 2020, 2021a, b, c, d, 2022a, b). By contrast, the Japanese Archipelago harbors 20 *Trachys* and 12 *Habroloma* species (Buprestidae: Tracheini) (Ohmomo and Fukutomi 2013), and no new taxa have been added since the monograph by Kurosawa (1959).

Leaf-mining habits in Buprestidae are believed to have evolved from wood-boring habits (Frost 1924), and the switch from wood-boring to leaf-mining has occurred several times in Buprestidae (Evans et al. 2015). The leaf-mining habits of Japanese trachyine species are characterized by full-depth blotch mining of the leaves of woody plants such as *Malus*, *Rosa*, *Prunus*, *Ulmus*, *Zelkova*, *Aphananthe*, *Broussonetia*, *Quercus*, *Castanopsis*, *Platycarya*, *Salix*, and *Deutzia*, or subwoody climbing plants such as *Pueraria*, *Amphicarpaea*, and *Desmodium* (Yano 1952), while in Europe many leaf-mining trachyine species are associated with herbaceous plants such as *Fragaria*, *Potentilla*, *Scabiosa*, *Stachys*, *Malva* (Frost 1924), and *Geranium* (Schaefer 1950).

Of the 32 Japanese trachyine species, host plants have been reported for 28 (Ohmomo and Fukutomi 2013). The known host plants belong to seven angiosperm orders: Fabales (3 spp.), Rosales (13. spp.), Fagales (5 spp.), Malpighiales (2 spp.), Malvales (1 sp.), Cornales (2 spp.), and Ericales (2 spp.). The host plant records are based mainly on observations of adult beetles feeding, with immature stages and leaf mines being reported only for nine *Trachys* and four *Habroloma* species in Japan (Yano 1952).

To shed light on the diversity and host plant associations of trachyine species in Japan, we have conducted extensive rearing of leaf-mining larvae on diverse plants and a substantial collection of mined leaves. From the accumulated materials, we identified two undescribed trachyine species. The two new species are associated with two new plant orders and families for Tracheini: Oxalidales (Elaeocarpaceae) and Santalales (Loranthaceae). Furthermore, we detected leaf mines for 31 trachyine species, including new leaf mine records for 18 trachyine species. In this paper, we describe the two new species, as well as the leaf mines of 31 trachyine species, and discuss the diversity and evolution of plant utilization patterns of trachyine species in the Japanese Archipelago.

## ﻿Materials and methods

We have conducted extensive sampling of buprestid leaf mines from the Japanese Archipelago since the 1980s. By rearing the leaf-mining larvae, we obtained 400 adult buprestid beetles. All of the specimens were collected by MK unless otherwise noted. The leaves containing leaf mines were dried, and the dried herbarium specimens have been deposited in the Kyoto University Museum (**KUM**).

The morphology of adult specimens was examined under a microscope (VHS-7000; Keyence). Specimens were photographed by synthesizing virtual images from a sequence of corresponding depth images. To observe male genitalia, the specimens were macerated in hot water and dissected under a microscope. The abdomen was removed from the body and then cleaned in 5% KOH solution for ~ 12 h at room temperature. After washing in distilled water, the terminalia extracted from the abdomen were mounted on slides with glycerol.

## ﻿Results

### ﻿Systematics of Japanese *Habroloma* Thomson, 1864

Among adult beetles that emerged from collected leaf mines, we identified two new *Habroloma* species. We describe the two species using the following key to Japanese species. In this key, *Habrolomahikosanensis* is missing because it is within the morphological variation of *Habrolomayuasai*.

### ﻿Key to the Japanese *Habroloma* Thomson, 1864 species

**Table:** 

1	Pronotum with a large distinct fovea at the post-inferior side of each anterior angle	***bifrons* (Kiesenwetter, 1879)**
–	Pronotum with a shallow depression at the post-inferior side of each anterior angle	**2**
2	Ventral surface of body flattened; thickness index (body thickness/body length) less than 0.36 (Fig. 2A–D); prosternal process inverted trapezoid, posterior margin linearly truncated (Fig. 3A–D)	**3**
–	Ventral surface of body convex ventrally; thickness index (body thickness/body length) greater than 0.37; prosternal process round or lingulate, longer than wide, posterior margin often rounded (Fig. 3E–J)	**6**
3	Elytra clothed with greyish golden hairs except silvery vitta (Fig. 1A, B)	**4**
–	Elytra clothed with greyish hairs except silvery vitta (Fig. 1C, D)	**5**
4	Elytra with a distinct V-shaped silvery vitta (Fig. 1B)	***eximium* (Lewis, 1892) [host: *Symplocos***]
–	Elytra without a distinct V-shaped silvery vitta (Fig. 1A)	***liukiuensis* Obenberger, 1940 [host: *Symplocos***]
5	Elytra with a V-shaped silvery vitta, which neighboring a V-shaped black vitta ahead (Fig. 1C); prosternal process slightly expanded posteriorly (Fig. 3C)	***griseonigra* (E. Saunders, 1873) [host: *Quercus***]
–	Elytra with two waving transverse silvery vittae on posterior half; anterior vitta M-shaped (Fig. 1D); prosternal process strongly expanded posteriorly (Fig. 3D)	***elaeocarpusi* Kato, sp. nov. [host: *Elaeocarpus***]
6	Basal 2/3 of elytra with a steel-blue patch (Fig. 1E)	***lewisii* (E. Saunders, 1873) [host: *Rosa***].
–	Elytra without steel-blue patch	**7**
7	Elytra with three wavy silvery and golden transverse vittae on posterior 2/3 (Fig. 1F); prosternal process round (Fig. 3F)	***taxillusi* Kato, sp. nov. [host: *Taxillus***]
–	Elytra with two wavy silvery or golden transverse vittae on posterior half (Fig. 1G–K); prosternal process lingulate, longer than wide (Fig. 3G–J)	**8**
8	Body subovate, less attenuated posteriorly, margins nearly parallel in basal half (Fig. 1G); prosternal process narrowest at base, with rounded posterior margin (Fig. 3G)	***nixilla*** (Obenberger, 1929) [**host: *Lagerstroemia***]
–	Body cuneiform, attenuating toward posterior end even from basal half (Fig. 1H–K); prosternal process with linearly truncated posterior margin (Fig. 3H–J)	**9**
9	Anterior wavy transverse silverly/golden band of posterior elytra complete (Fig. 1H, I); prosternal process with posterior margin linearly truncated (Fig. 3H, I)	**10**
–	Anterior wavy transverse silverly/golden band of posterior elytra disconnected midway (Fig. 1J, K); prosternal process with posterior margin arched (Fig. 3J)	**12**
10	Anterior wavy transverse band silvery (Fig. 1I)	***marginicolle* (Fairmaire, 1888) [host: *Rubus***]
–	Anterior wavy transverse band grayish-golden and partly silvery (Fig. 1H)	**11**
11	Elytra with the sides constricted behind humeri; prosternal process as long as wide	***asahinai* Y. Kurosawa, 1959 [host: *Rubus***]
–	Elytra with the sides not constricted behind humeri (Fig. 1H); prosternal process longer than wide (Fig. 3H)	***yuasai* Y. Kurosawa, 1976 [host: *Platycarya***]
12	Elytra strongly attenuate from base to the apex, with the sides less arcuate and distinctly constricted behind humeri (Fig. 1J)	***subbicorne* (Motschulsky, 1860) [host: *Rubus***]
–	Elytra attenuate from the base to the apex, with the sides not constricted behind humeri (Fig. 1K)	***atronitidum* (Gebhardt, 1929) [host: *Rubus***]

#### ﻿ Habroloma elaeocarpusi sp. nov.

Taxon classificationAnimaliaColeopteraBuprestidae

EBB90B02-CB04-5F63-B910-5B648D61476B

https://zoobank.org/13395C99-4CB0-48AB-8BAA-5BA2C4799DEF

**Figure 1.**
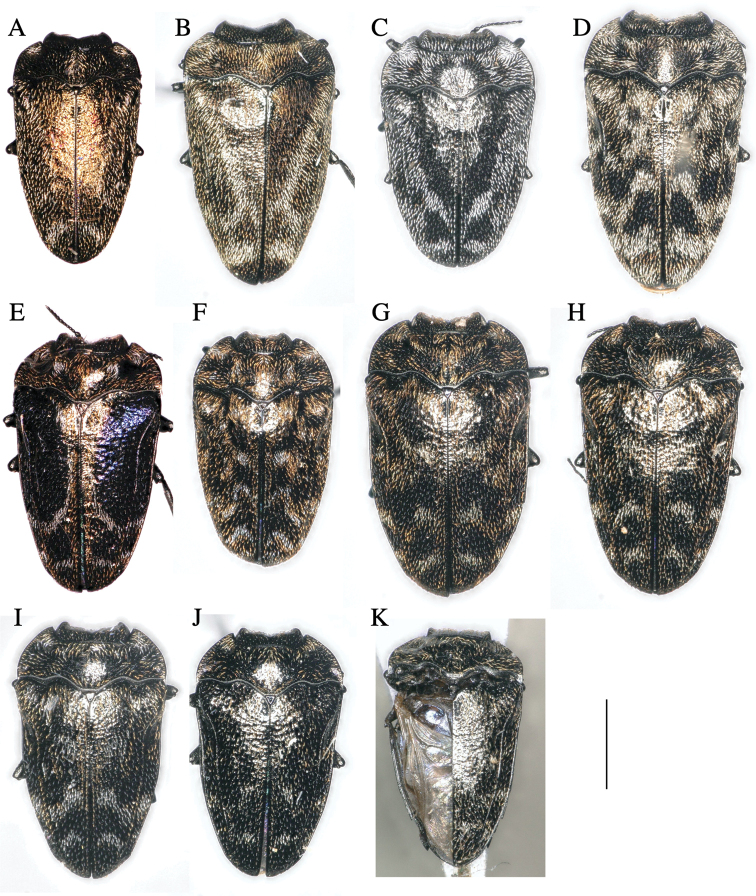
*Habroloma* species of Japan, adult dorsal views **A***H.liukiuense***B***H.eximiumeupoetum***C***H.griseonigrum***D***H.elaeocarpusi***E***H.lewisii***F***H.taxillusi***G***H.nixillainsulicola***H***H.yuasai***I***H.marginicolle***J***H.subbicorne***K***H.atronitidum*. Scale bar: 1 mm.

**Figure 2.**
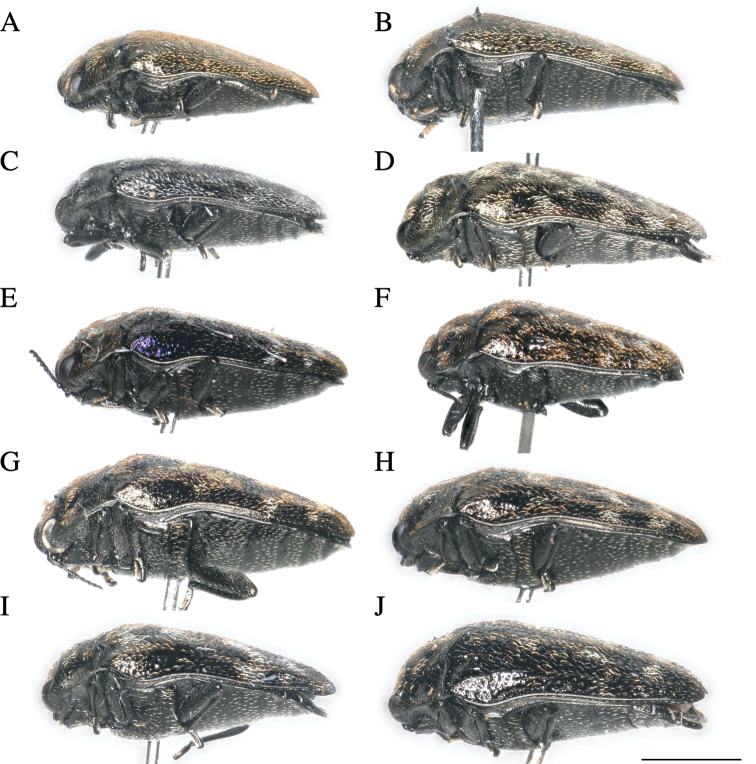
*Habroloma* species of Japan, adult lateral views **A***H.liukiuense***B***H.eximiumeupoetum***C***H.griseonigrum***D***H.elaeocarpusi***E***H.lewisii***F***H.taxillusi***G***H.nixillainsulicola***H***H.yuasai***I***H.marginicolle***J***H.subbicorne*. Scale bar: 1 mm.

**Figure 3.**
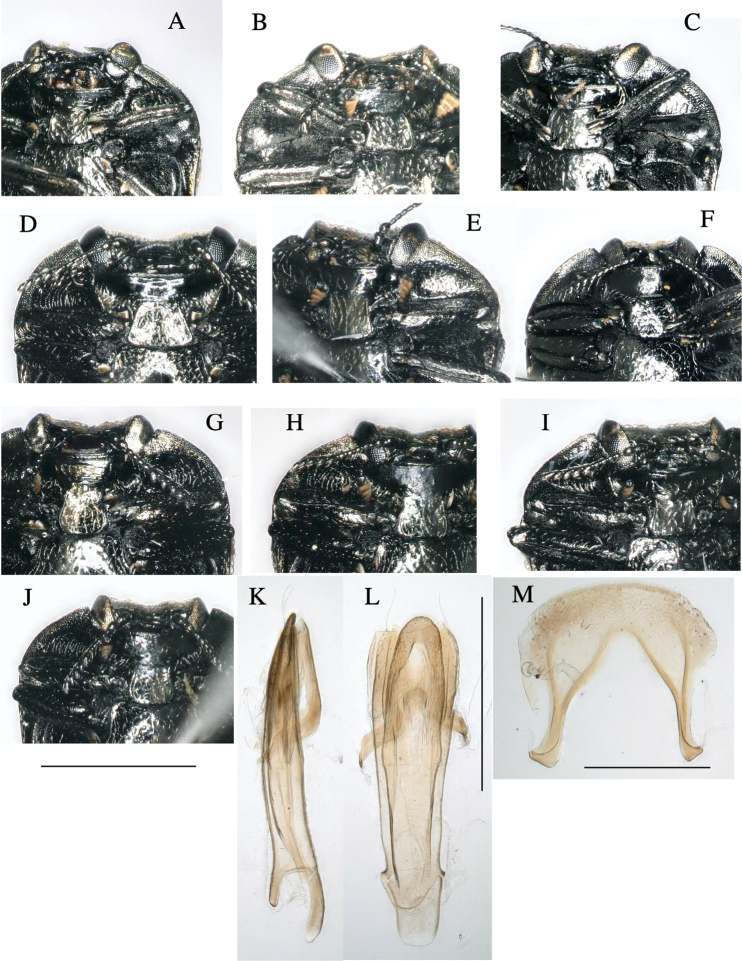
*Habroloma* species of Japan, adult ventral views (**A–J**) and male genitalia of *H.elaeocarpusi* (**K–M**). **A***H.liukiuense***B***H.eximiumeupoetum***C***H.griseonigrum***D***H.elaeocarpusi***E***H.lewisii***F***H.taxillusi***G***H.nixillainsulicola***H***H.yuasai***I***H.marginicolle***J***H.subbicorne***K, L** lateral and ventral views of tegmen, pennis and sternite IX **M** tergite VIII. Scale bars 1 mm (**A–J**); 0.5 mm (**K–M**).

Figs 1D , 2D , 3D, K–M

##### Material examined.

***Holotype***: Japan: ♂ (MK-BP-a327), Mt. Osuzu, Tsuno-cho, Miyazaki Pref. (32.262°N, 131.471°E, 230 m above sea level), 14-VII-2021 (as larva on *Elaeocarpusjaponicus*), emerged on 27-VII-2021, NSMT-I-C-200265.

***Paratypes***: Japan: 1♂(MK-BP-a360), same data as holotype, emerged on 30-VII-2021, NSMT-I-C-200266; 1♀ (MK-BP-k35), Isso, Yakushima-cho, Yaku Island (30.440°N, 130.472°E, 60 m above sea level), 11-VI-1993 (as larva on *Elaeocarpusjaponicus*), emerged on 26-VI-1993, NSMT-I-C-200267.

##### Other material.

Japan: 2♂2♀, same data as holotype, emerged on 27-VII–2-VIII-2021.

##### Diagnosis.

A small wedge-shaped species (length 3.1–3.3 mm) having pronotum with posterior margin trisinuate. Elytra rather flattened, ornamentation consisting of white pubescence; on posterior half with three transverse bands, anterior one obliquely zigzag, two posterior ones transversely straight. Male genitalia with slender tegmen with paramere setiferous on anterior margin and slender pennis with rounded apex. Larvae mine leaves of *Elaeocarpusjaponicus*.

##### Description.

**Adult male**: (Figs 1D, 2D, 3D) ***Body*** somewhat wedge-shaped and attenuated posteriorly; above entirely black-aeneous; body beneath, legs, and antennae black, with a very slight aeneous tinge, except tarsal lamellae brownish.

***Head***, seen from above, transverse, broadly and sharply excavated between the eyes, with the inferior rim of the eyes strongly and rather suddenly produced; frons with the median impression distinct; fovea just above each antennal cavity obsolete and indistinct; surface rather smooth, sparsely scattering laterally with traces of variolate and ocellate punctures, and sparsely clothed with whitish recumbent hairs; clypeal suture transverse, somewhat arcuate exteriorly; clypeus transverse, ~ 2.6× as wide as long, with the anterior margin somewhat arcuately emarginate; antennal cavities surrounded posteriorly with elevated carina; antennae short and compact, with the third segment ~ 1.5× as long as the fourth, with apical five segments serrated.

***Pronotum*** transverse, widest just before the base, distinctly wider than elytra, and ~ 3.2× as wide as long; sides slightly but distinctly expanded just before the base, then crescent-shaped and strongly attenuated to the anterior angles, which are acute and strongly produced in dorsal aspect; anterior margin deeply, broadly and arcuately emarginate; posterior margin trisinuate, produced and subtruncate, narrowly and slightly emarginate just before scutellum; posterior angles acute and produced posteriorly; disk dilated laterally, broadly and obsoletely depressed at the anterior half of the lateral dilation on each side, but without fovea, and obsoletely impressed along the basal lobe causing the middle of the disk to be somewhat convex; surface lustrous, punctured with traces of large, obsolete, shallow, somewhat ocellate punctures, and sparsely clothed with whitish hairs. Scutellum smooth and triangular.

***Elytra*** rather deplanate, widest at the base, ~ 1.3× as long as wide and ~ 4.3× as long as pronotum; sides feebly sinuate and narrowed or subparallel to the anterior 2/5, and then arcuately attenuated to the apex, but the attenuation somewhat angulate near the apex; sutural margin not elevated entirely; humeri slightly prominent; basal depressions along the base transverse; lateral carinae subparallel to the lateral margin; disk constricted behind humeri, narrowly and obsoletely impressed along the inferior side of each lateral carina; surface rather uniformly but coarsely punctate with shallow, ill-defined, irregularly sized punctures, with the punctuation being somewhat rugous at the sides; ornamentation consisting of white, yellowish-grey, and blackish hairs, with the whitish hairs being predominant. Ornamentation consisting of white pubescence arranged on each elytron as follows: at base with two irregular spots, at mid length near suture with one irregular spot, toward side with one narrow, wavy, and irregular strip, on posterior half with three transverse bands, anterior one obliquely zigzag, two posterior ones transversely straight.

***Body*** beneath scattered with very fine inconspicuous cinereous hairs. Prosternal process inverted trapezoidal, narrow toward the base, ~ 1.3× broader than long, with the apex almost truncate. Metasternum slightly convex coarsely punctate with variolate and obsolete punctures at the middle. Abdomen beneath rather uniformly punctate with shallow, obsolete variolate punctures. Legs normal; posterior coxae depressed entirely, with the latero-posterior angles acute and produced latero-posteriorly.

***Male genitalia*** (Fig. 3K–M). Sternite VIII wide, roundly arcuated along anterior margin, furnished with several setae on each side of anterior margin. Tegmen slender; paramere setiferous on anterior margin; phallobase wide, ~ 1/5 length of tegmen. Penis slender, slightly shorter than tegmen; round at apex, basally with median struts ~ 1/3 length of penis.

**Female**. Like the male, but more robust. Length: 3.1–3.3 mm, width: 1.8–1.9 mm.

##### Etymology.

The name indicates the host plant genus, *Elaeocarpus*.

##### Japanese name.

Kobanmochi-hiratachibi-tamamushi.

##### Host plant.

*Elaeocarpusjaponicus* Sieb. et Zucc.

##### Habitat.

Primary evergreen forests dominated by Castanopsissieboldiisubsp.sieboldii.

##### Distribution.

Japan (Kyushu and Yaku Island).

#### ﻿ Habroloma taxillusi sp. nov.

Taxon classificationAnimaliaColeopteraBuprestidae

20A5AECF-8D61-5D89-B4FA-965023FDA3DC

https://zoobank.org/3A0F84C8-254D-4F21-AD10-90501E54DB37

Figs 1F , 2F , 3F

##### Material examined.

***Holotype***: Japan: ♂ (MK-BP-k40), Yakukachi, Amami-shi, Kagoshima Pref. (28.228°N, 129.347°E, 40 m above sea level), 23-V-2009 (as larva on *Taxillusyadoriki* collected by A. Kawakita), emerged on 7-VI-2009, NSMT-I-C-200268.

***Paratype***: Japan: 1♀(MK-BP-k39), same data as holotype, emerged on 2-VI-2009, N NSMT-I-C-200269.

##### Diagnosis.

A small wedge-shaped species (length 2.5–2.7 mm) having pronotum with posterior margin trisinuate. Elytra slightly convex around base, ornamentation consisting of yellowish-grey pubescence; on posterior 2/3 with three transverse bands, first two obliquely zigzag, apical one slightly transversely waved. Larvae mine leaves of a mistletoe species, *Taxillusyadoriki*.

##### Description.

**Adult male**: (Figs 1F, 2F, 3F) ***Body*** somewhat wedge-shaped and attenuated posteriorly; above entirely black-aeneous; body beneath, legs, and antennae black, with a very slight aeneous tinge, except tarsal lamellae dark brownish.

***Head***, seen from above, transverse, broadly excavated between the eyes, with the inferior rim of the eyes strongly produced; frons with the median impression distinct; fovea just above each antennal cavity obsolete and indistinct; surface rather smooth, sparsely scattered laterally with traces of variolate and ocellate punctures, and sparsely clothed with recumbent yellowish-grey hairs; clypeal suture transverse, somewhat arcuate exteriorly; clypeus transverse, ~ 2.6× as wide as long, with the anterior margin somewhat arcuately emarginate; antennal cavities surrounded posteriorly with elevated carina; antennae short and compact, with the third segment ~ 1.5× as long as the fourth, and five apical serrated segments.

***Pronotum*** transverse, widest just before the base, as wide as elytra, and ~ 2.4× as wide as long; sides slightly but distinctly expanded just before the base, then crescent-shaped and strongly attenuated to the anterior angles, which are acute and strongly produced in dorsal aspect; anterior margin deeply, broadly, and arcuately emarginate; posterior margin trisinuate and subtruncate, narrowly and slightly emarginate just before scutellum; posterior angles acute and produced posteriorly; disk dilated laterally, broadly and obsoletely depressed at the anterior half of the lateral dilation on each side, but without fovea, and obsoletely impressed along the basal lobe causing the middle of the disk to be somewhat convex; surface lustrous, punctured the traces of large, obsolete, shallow, somewhat ocellate structures, and sparsely clothed with yellowish gray hairs. Scutellum smooth and triangular.

***Elytra*** slightly convex along base, widest at the base, ~ 1.4× as long as wide and ~ 3.3× as long as pronotum; sides feebly sinuate and narrowed or subparallel to the anterior 2/5, and then arcuately attenuated to the apex but with the attenuation somewhat angulate near the apex; humeri slightly prominent; basal depressions along the base transverse; lateral carinae subparallel to the lateral margin; disk constricted behind humeri, narrowly and obsoletely impressed along the inferior side of each lateral carina; surface rather uniformly but coarsely punctate with shallow, ill-defined, irregularly sized punctures, but the punctuation somewhat rugous at the sides. Ornamentation consisting of yellowish grey pubescence arranged on each elytron as follows: at base with two irregular spots, on posterior 2/3 with three transverse bands, first and second ones obliquely zigzag, apical one slightly transversely waved; with transverse irregular spot apically.

***Body*** beneath scattered with very fine inconspicuous cinereous hairs. Prosternal process rounded, ~ 1.27× broader than long. Metasternum slightly convex and coarsely punctate, with variolate and obsolete punctures at the middle. Abdomen beneath rather uniformly punctate with shallow, obsolete variolate punctures. Legs normal; posterior coxae depressed entirely, with the latero-posterior angles acute and produced latero-posteriorly.

***Male genitalia***: not studied.

**Female**. Like the male, but more robust. Ornamentation of elytra is similar but pubescence more whitish in female. Body length: 2.5–2.7 mm, width: 1.5–1.7 mm.

##### Etymology.

The specific name indicates host plant genus, *Taxillus*.

##### Japanese name.

Obayadorigi-hiratachibi-tamamushi.

##### Host plant.

*Taxillusyadoriki* (Maxim.) Danser [Loranthaceae]

##### Habitat.

Canopy of primary evergreen forests dominated by Castanopsissieboldiisubsp.lutchuensis (Fig. 5L).

##### Distribution.

Japan (Amami-Oshima Island, known only from the type locality).

###### Leaf mines of Japanese Tracheini species

Leaf mines of Tracheini species have the following characteristics. The mined leaves are mature leaves that have completed expansion and hardening. An egg is laid on the upper side of a leaf and covered by a circular brown glossy coating, which is secreted by an adult female. Pupation takes place within the mine. Hereafter, we describe leaf mines of the 14 *Habroloma* and 20 *Trachys* species in Japan.

### ﻿*Habroloma* species checklist

**1. *Habrolomasubbicorne* (Motschulsky, 1860)**

Fig. 4A–E

**Host plant**. Rosaceae: *Rubusparvifolius* (Yano 1952), *Rubuspalmatus*, *Rubusbuergeri* (Kurosawa 1959).

**Leaf mine.** Brown full-depth blotch mine on mature leaf. Egg is laid at a distance from leaf margin and the mine expands in the leaf blade. Frass is thread-like.

**Figure 4.**
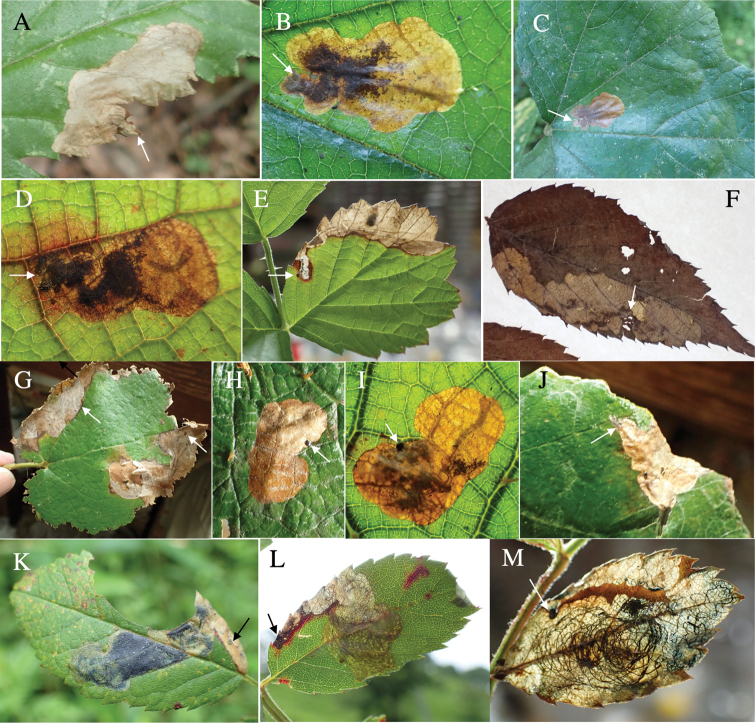
Leaf mines of *Habroloma* spp. on leaves of Rosaceae**A–E***H.subbicorne* on Rubuspalmatusvar.coptophyllus (**A**), *Rubusbuergeri* (**B–D**), and *Rubusparvifolius* (**E**) **F***H.atronitidum* on *Rubusvernus***G–I***H.marginicolle* on *Rubussieboldii***J***H.asahinai* on *Rubussieboldii***K–M***H.lewisii* on *Rosamultiflora*. Arrows indicate oviposition scars.

**Material examined.** Nishihoragawa, Kiso, Nagano Pref. 6-VIII-2015 (as larva on Rubuspalmatusvar.coptophyllus), emerged on 27-VIII-2015 (Fig. 4A); Sakai-gawa, Takaoka, Miyazaki, Miyazaki Pref., 21-IX-2020 (as larva on *R.buergeri*), emerged on 7-VII-2020 (Fig. 4B, C); Seikandoro, Kumanogawa, Shingu, Wakayama Pref., 14-VII-2021 (as larva on *R.buergeri*), emerged on 31-VII-2021 (Fig. 4D); Furubokke, Kasai, Hyogo Pref., 11-IX-2018 (as pupa on *R.parvifoliusparvifolius*), emerged on 15-IX-2018 (Fig. 4E).

**2. *H.atronitidum* (Gebhardt, 1929)**

Fig. 4F

**Host plant**. Rosaceae: *Rubusvernus* (new record).

**Leaf mine.** Brown full-depth blotch mine on mature leaf. Egg is laid near leaf margin, and the mine expands along the leaf margin. Frass is thread-like.

**Material examined.** Mumyo-dani, Niimi, Okayama Pref., 9-VII-1991 (as pupa on *Rubusvernus*), emerged on ?-VIII-1991 (Fig. 4F).

**3. *H.marginicolle* (Fairmaire, 1888)**

Fig. 4G–I

**Host plant**. Rosaceae: *Rubussieboldii* (Kurosawa 1959), *Rubusbuergeri* (new record).

**Leaf mine.** Brown blotch mine on mature leaf. Egg is laid at a distance from leaf margin, and the mine expands in the leaf blade. Frass is thread-like.

**Material examined.** Modo, Minamata, Kumamoto Pref., 26-V-2018 (vacant mine of *Rubussieboldii*) (Fig. 4G); Inohae, Kitago, Nichinan, Miyazaki Pref., 16-VI-2019 (as larva on *Rubussieboldii*), emerged on 19-VII-2019; Inohae, Kitago, Nichinan, Miyazaki Pref., 16-VI-2019 (as larva on *R.buergeri*), emerged on 21-VII-2019 (Fig. 4H, I).

**4. *H.asahinai* Y. Kurosawa, 1959**

Fig. 4J

**Host plant**. Rosaceae: *Rubussieboldii* (Ohmomo and Fukutomi 2013).

**Leaf mine.** Brown full-depth blotch mine on mature leaf. Egg is laid near leaf margin, and the mine expands along the leaf margin. Frass is thread-like.

**Material examined.** Okuma, Kunigami, Okinawa Pref., 30-III-2018 (vacant mine of *Rubussieboldii*) (Fig. 4J).

**5. *H.lewisii* (E. Saunders, 1873)**

Fig. 4K–M

**Host plant**. Rosaceae: *Rosamultiflora* (Yano 1952).

**Leaf mine.** Brown, full-depth sometimes bluish, linear-blotch mine on mature leaf. Egg is laid along leaf margin, and the mine expands along leaf margin. Frass is thread-like, coiling or undulating for an extended length.

**Material examined.** Sabushi-gawa, Niimi, Okayama Pref., 1-VII-2018 (as larva on *Rosamultiflora*), emerged on 18-VII-2018 (Fig. 7K); Shimotokuyama, Hiruzen, Maniwa, Okayama Pref., 1-VII-2018 (as larva on *Rosamultiflora*) (Fig. 4L, M).

**6. *H.griseonigrum* (E. Saunders, 1873)**

Fig. 5A

**Host plant**. Fagaceae: *Quercusglauca*, *Q.acutissima*, *Q.serrata* (Yano 1952), *Quercusacuta* (Ohmomo and Fukutomi 2013), *Quercushondae* (new record).

**Figure 5.**
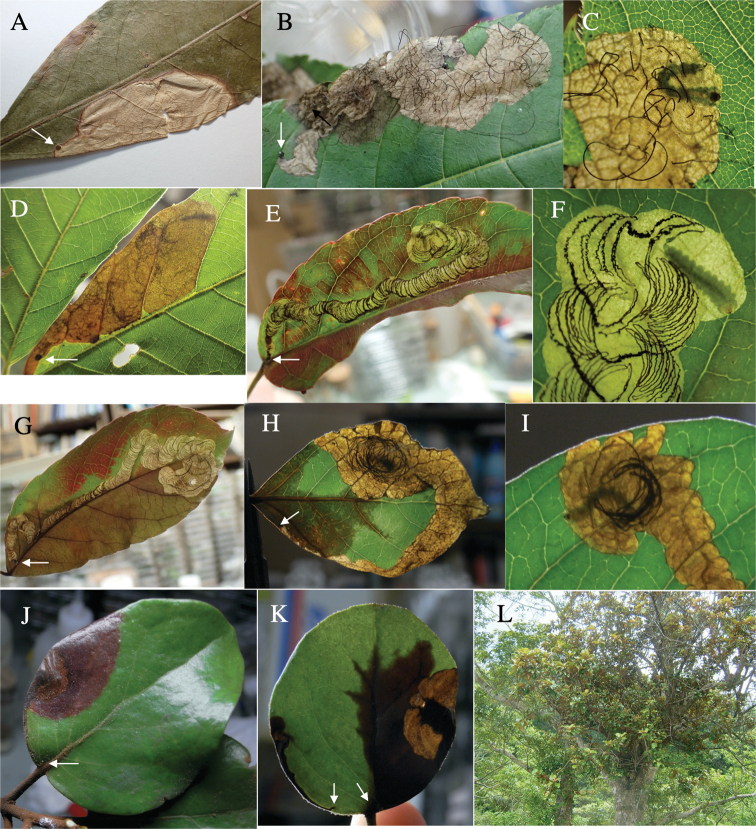
Leaf mines of *Habroloma* spp. on leaves of Fagaceae, Juglandaceae, Elaeocarpaceae, Lythraceae, and Loranthaceae**A***H.griseonigrum* on *Quercushondae***B–D***H.yuasai* on *Platycaryastrobilacea***E–G***H.elaeocarpusi* on *Elaeocarpusjaponicus***H, I***H.nixilla* on *Lagerstroemiasubcostata***J–L***H.taxillusi* on *Taxillusyadoriki*. Arrows indicate oviposition scars.

**Leaf mine.** Brown full-depth blotch mine on mature leaf. Egg is laid near leaf margin of leaf base, and the mine expands upwards along leaf margin. Frass is thread-like.

**Material examined.** Gion, Yayoi, Saeki, Ooita Pref., 14-VI-1998 (as larva on *Quercushondae*); emerged on 8-VII-1998 (Fig. 5A).

**7. *H.yuasai* Y. Kurosawa, 1976**

Fig. 5B–D

**Host plant**. Juglandaceae: *Platycaryastrobilacea* (Yano 1952).

**Leaf mine.** Brown full-depth linear-blotch mine on mature leaf. Egg is laid near leaf margin, and the mine expands along leaf margin. Frass is thread-like, excreted from the mine through cracks of upper epidermis.

**Material examined.** Makido, Niimi, Okayama Pref., 22-VI-2020 (as larva on *Platycaryastrobilacea*), emerged on 20-VII-2020 (Fig. 5B–D).

**8. *H.elaeocarpusi* sp. nov**

Fig. 5E–G

**Host plant**. Elaeocarpaceae: *Elaeocarpusjaponicus* (new record).

**Leaf mine.** Pale brown full-depth linear-blotch mine on mature leaf. Egg is laid just beside midrib of leaf base, and the mine expands along midrib or along leaf margin. Frass is thread-like; frass line iterating arc-shaped reciprocating motion.

**Material examined.** Mt. Osuzu, Tsuno-cho, Miyazaki Pref., 14-VII-2021 (as larva on *Elaeocarepusjaponicus*), emerged on 27-VII-2021 (Fig. 5E–G).

**9. *H.bifrons* (Kiesenwetter, 1879)**

**Host plant**. Unknown, while related species in Europe is associated with *Geranium* (Geraniaceae).

**Leaf mine.** Unknown.

**10. *H.nixillainsulicola* Y. Kurosawa, 1959**

Fig. 5H, I

**Host plant**. Lythraceae: *Lagerstroemiasubcostata* (Kurosawa 1959).

**Leaf mine.** Brown full-depth linear-blotch mine on mature leaf. Egg is laid along leaf margin of leaf base, and the mine expands along leaf margin. Frass is thread-like, coiling in the mine.

**Material examined.** Sumiyo, Amami, Kagoshima Pref., 23-V-2009 (as larva on *Lagerstroemiasubcostata*), emerged on 6-VI-2009 (Fig. 8H, I).

**11. *H.taxillusi* sp. nov.**

Fig. 5J–L

**Host plant**. Loranthaceae: *Taxillusyadoriki* (new record).

**Leaf mine.** Brown full-depth linear-blotch mine on mature leaf. Egg is laid along leaf margin of leaf base, and the mine expands along leaf margin. Frass is granular, accumulated in the center of the mine.

**Material examined.** Yakukachi, Amami-shi, Kagoshima Pref., 23-V-2009 (as larva on *Taxillusyadoriki*), emerged on 7-VI-2009 (Fig. 5J–L).

**12a. *H.eximiumeximium* (Lewis, 1893)**

**Host plant**. Symplocaceae: *Symplocoslancifolia* (Kurosawa 1959).

**Leaf mine.** Unknown.

**12b. *H.eximiumeupoetum*** (Obenberger, 1929)

Fig. 6A–C

**Host plant**. Symplocaceae: *Symplocosprunifolia* (Kurosawa 1976), while the identification seems to be incorrect; *S.caudata* (new record).

**Leaf mine.** Pale brown full-depth linear-blotch mine on mature leaf. Egg is laid near midrib of leaf base, and the hatched larva enters midrib and bores into petiole, causing the leaf to fall off from the branch by being abscised at the petiole base. After the leaf-fall, the mine departs from the midrib and slowly expands upwards along leaf margin or along midrib. After advancing halfway, the mine abruptly expands to become a blotch mine. Frass is thread-like, going in a zigzag in the early linear mine, and becomes thick cord-like without undulating as the mine expands. The fallen leaf is kept green for ca. two weeks, during which the larva completes its development.

**Figure 6.**
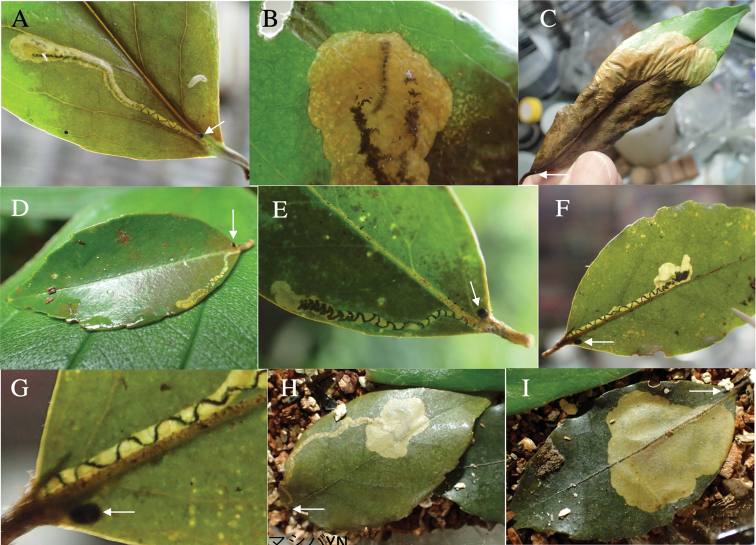
Leaf mines of *Habroloma* spp. on leaves of Symplocaceae**A–C***H.eximium* on *Symplocoscaudata***D–I***H.liukiuense* on *Symplocosmicrocalyx*. Arrows indicate oviposition scars.

**Material examined.** Komi, Iriomote Is., Yaeyama, Okinawa Pref., 10-V-2020 (as larva on *Symplocoscaudata*), emerged on 15-VI-2020 (Fig. 6A–C).

**13. *H.liukiuense* (Obenberger, 1940)**

Fig. 6D–I

**Host plant**. Symplocaceae: *Symplocosokinawensis* (Ohmomo and Fukutomi 2013), *S.microcalyx* (Ohmomo and Fukutomi 2013).

**Leaf mine.** Pale brown full-depth linear-blotch mine on mature leaf. Egg is laid near midrib of leaf base, and the hatched larva enters midrib and bores into petiole, causing the leaf to fall off from the branch by being abscised at the petiole base. After the leaf-fall, the mine departs from the midrib and slowly expands upwards along leaf margin or along midrib. After advancing halfway, the mine abruptly expands to become a blotch mine. Frass is thread-like, going in a zigzag in the early linear mine, and becomes granular. The fallen leaf is kept green for ca. two weeks, during which the larva completes its development.

**Material examined.** Foothill of Mt. Yonaha, Kunigami, Okinawa Pref., 6-VI-2018 (as larva on *Symplocosmicrocalyx*), emerged on 12-VII-2018 (Fig. 6D–I).

**14. *H.hikosanense* Y. Kurosawa, 1959**

**Host plant**. Unknown.

**Leaf mine.** Unknown.

### ﻿*Trachys* species checklist

**1. *Trachysauricollis* E. Saunders, 1873**

Fig. 7A

**Host plant.**Fabaceae: Puerariamontanavar.lobata (Yano 1952).

**Leaf mine.** Gray full-depth blotch mine on mature leaflet. Egg is laid in an inner area of leaf blade, and mine expands toward leaf margin. Frass is granular and distributed all over the mine.

**Material examined**. Mt. Osuzu, Tsuno, Miyazaki Pref., 14-VII-2021 (as larva), emerged on 30-VII-2021 (Fig. 3A).

**2. *Trachysreitteri* Obenberger, 1930**

Fig. 7B–D

**Host plant**. Fabaceae: *Amphicarpaeaedgeworthii* (Yano 1952), Puerariamontanavar.lobata, *Rhynchosiavolubilis*, *Glycinemax* (Ohmomo and Fukutomi 2013), *Glycinesoja* (new record).

**Leaf mine.** White full-depth blotch mine on mature leaflet. Egg is laid along anterior margin of a leaflet, and the mine expands toward leaf base. Frass is granular and distributed all over the mine.

**Figure 7.**
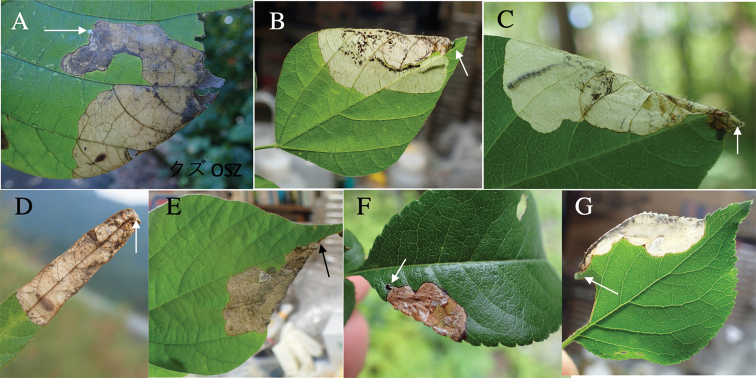
Leaf-mines of *Trachys* spp. on leaves of Fabaceae and Rosaceae**A***T.auricollis* on Puerariamontanavar.lobata**B–D***T.reitteri* on *Amphicarpaeaedgeworthii* (**B, C**) and *Glycinesoja* (**D**) **E***T.tokyoensis* on Desmodiumpodocarpumsubsp.fallax**F***T.toringoi* on *Chaenomelesjaponica***G***T.inconspicuus* on *Prunusmume*. Arrows indicate oviposition scars.

**Materials examined.** Kiso-Fukushima, Nagano Pref., 10-VIII-2019 (as larva on *A.edgeworthii*), emerged on 23-VII-2019 (Fig. 7B) Hirogawara, Yamanashi Pref., 30-VII-2018 (as larva on *A.edgeworthii*) (Fig. 7C); Kawaguchi-ko Lake, Yamanashi Pref., 20-IX-2014 (as pupa on *G.soja*), emerged on 1-X-2014 (Fig. 7D).

**3. *Trachystokyoensis* Obenberger, 1940**

Fig. 7E

**Host plant.**Fabaceae: Desmodiumpodocarpumsubsp.oxyphyllum (Yano 1952), Desmodiumpodocarpumsubsp.fallax (new record).

**Leaf mine.** Brown full-depth blotch mine on mature leaflet. Egg is laid along anterior margin of a leaflet, and the mine expands toward leaf base. Frass is granular and distributed all over the mine.

**Material examined.** Kawaguchi-ko Lake, Yamanashi Pref., 19-IX-2017 (as pupa on Desmodiumpodocarpumsubsp.fallax), emerged on 10-X-2017 (Fig. 7E).

**4. *Trachystoringoi* Y. Kurosawa, 1951**

Fig. 7F

**Host plant**. Rosaceae: *Chaenomelesjaponica*, *Malussieboldii* (Yano 1952), *Cydoniaoblonga*, *Maluspumila*, *Pyruspyrifolia*, *Amelanchierasiatica* (Kurosawa 1959).

**Leaf mine.** Brown blotch mine on mature leaf. Egg is laid in an inner basal area of leaf blade, and the mine expands toward leaf top. Frass is granular and distributed all over the mine.

**Material examined.** Hara-mura, Suwa, Nagano Pref., 15-VIII-2018 (as vacant mine of *Chaenomelesjaponica*) (Fig. 7F).

**5. *Trachysinconspicuus* E. Saunders, 1873**

Fig. 7G

**Host plant**. Rosaceae: *Prunusmume*, *P.salicina* (Yano 1952).

**Leaf mine.** White full-depth blotch mine on mature leaf. Egg is laid along leaf margin, and the mine expands along leaf margin. Frass is granular and distributed all over the mine.

**Material examined.** Kamikoma, Yamashiro, Kizugawa, Kyoto Pref., 17-VI-2016 (as pupa on *Prunusmume*), emerged on 19-VI-2016 (Fig. 7G).

**6. *Trachyspecirkai* Obenberger, 1925**

Fig. 8A

**Host plant**. Ulmaceae: Ulmusdavidianavar.japonica (Ohmomo and Fukutomi 2013).

**Leaf mine.** Brown full-depth blotch mine on mature leaf. Egg is laid near midrib, and the mine expands along lateral vein and then along leaf margin. Frass is granular and distributed all over the mine.

**Figure 8.**
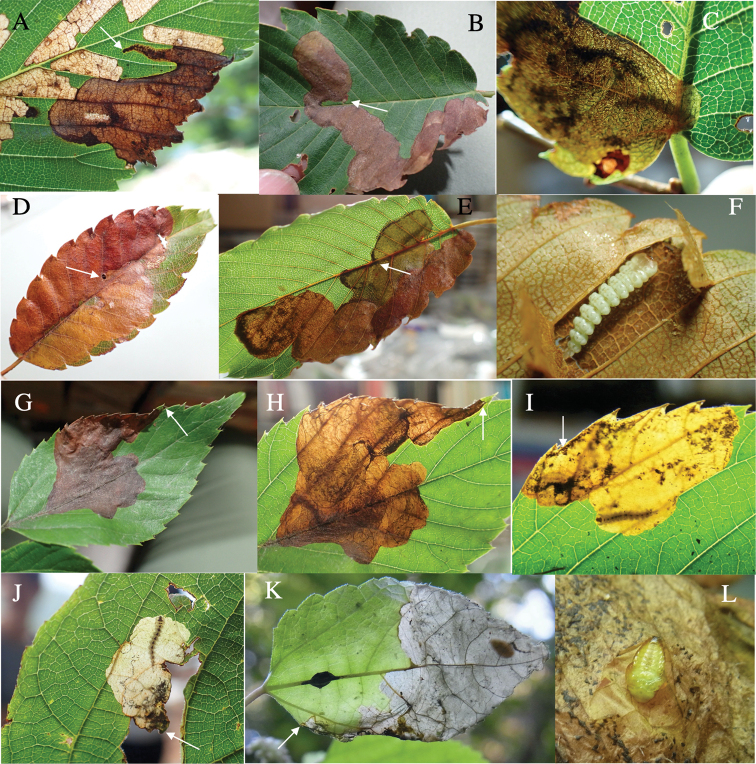
Leaf mines of *Trachys* spp. on leaves of Ulmaceae and Cannabaceae**A***T.pecirkai* on Ulmusdavidianavar.japonica**B–D***T.yanoi* on *Zelkovaserrata***E, F***T.cupricolor* on *Zelkovaserrata***G, H***T.griseofasciatus* on *Aphanantheaspera***I***T.ineditus* on *A.aspera***J–L***T.broussonetiae* on *Broussonetiakazinoki* (**J**) and *Fatouavillosa* (**K, L**). Arrows indicate oviposition scars.

**Material examined.** Suekawa, Kaida, Kiso, Nagano Pref., 5-VIII-2022 (as vacant mine of Ulmusdavidianavar.japonica) (Fig. 8A).

**7. *Trachyscupricolor* E. Saunders, 1873**

Fig. 8B, C

**Host plant**. Ulmaceae: *Zelkovaserrata* (Ohmomo and Fukutomi 2013).

**Leaf mine.** Dark brown full-depth blotch mine on mature leaf. Egg is laid along midrib, and the mine expands along leaf margin. Frass is granular and distributed all over the mine.

**Material examined.** Mt. Ibuki, Maibara, Shiga Pref., 29-VII-2020 (as pupa on *Zelkovaserrata*), emerged on 25-VIII-2020 (Fig. 8B, C).

**8. *Trachysyanoi* Y. Kurosawa, 1959**

Fig. 8D–F

**Host plant**. Ulmaceae: *Zelkovaserrata* (Kurosawa 1959).

**Leaf mine.** Brown full-depth blotch mine on mature leaf. Egg is laid along leaf margin, and the mine expands along leaf margin. Frass is granular and distributed all over the mine.

**Material examined.** Mt. Shizuhata, Aoi-ku, Shizuoka, Shizuoka Pref., 27-VI-2018 (as pupa on *Zelkovaserrata*), emerged on 12-VII-2018 (Fig. 8D–F).

**9. *Trachysgriseofasciatus* E. Saunders, 1873**

Fig. 8G, H

**Host plant**. Cannabaceae: *Aphanantheaspera* (Yano 1952), *Celtissinensis* (Ohmomo and Fukutomi 2013).

**Leaf mine.** Dark brown full-depth blotch mine on mature leaf. Egg is laid along anterior leaf margin, and the mine expands along leaf margin. Frass is thread-like and distributed all over the mine.

**Material examined.** Demachi-yanagi, Shimogamo, Sakyo, Kyoto Pref., 1-VII-2020 (as pupa on *Aphanantheaspera*), emerged on 7-VIII-2020 (Fig. 8G, H).

**10. *Trachysineditus* E. Saunders, 1873**

Fig. 8I

**Host plant**. Cannabaceae: *Aphanantheaspera* (Ohmomo and Fukutomi 2013).

**Leaf mine.** White full-depth blotch mine on mature leaf. Egg is laid along leaf margin, and the mine expands along leaf margin. Frass is granular and distributed all over the mine.

**Material examined.** Mukoujima, Uji, Kyoto Pref., 15-VII-2012 (as larva on *Aphanantheaspera*), emerged on 28-VII-2012 (Fig. 8I).

**11. *Trachysbroussonetiae* Y. Kurosawa, 1985**

Fig. 8J–L

**Host plant**. Moraceae: *Broussonetiakazinoki* (Yano 1952), *Fatouavillosa* (new record). The latter species is unique for host plants of Tracheini, because it is a herbaceous species.

**Leaf mine.** White full-depth blotch mine on mature leaf. Egg is laid along leaf margin, and the mine expands along leaf margin. Frass is granular and distributed all over the mine.

**Materials examined.** Chigonosawa, Kiso-fukushima, Kiso, Nagano Pref., 4-VIII-2018 (as larva on *Broussonetiakazinoki*), emerged on 30-VIII-2018 (Fig. 8J); Mumyo-dani, Niimi, Okayama Pref., 27-IX-2013 (as larva on *Fatouavillos*), emerged on 5-X-2013 (Fig. 8K, L).

**12. *Trachysvariolaris* E. Saunders, 1873**

Fig. 9A–C

**Host plant**. Fagaceae: *Quercusglauca*, *Q.serrata*, *Q.variabilis* (Yano 1952), *Q.hondae* (new record).

**Leaf mine.** Gray full-depth blotch mine on mature leaf. Egg is laid near leaf margin, and the mine expands along leaf margin. Frass is granular and distributed all over the mine.

**Figure 9.**
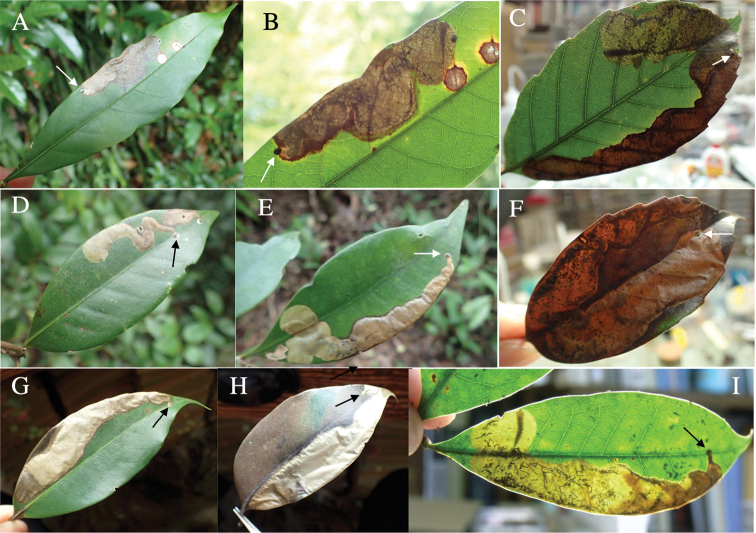
Leaf mines of *Trachys* spp. on leaves of Fagaceae**A–C***T.variolaris* on *Quercushondae* (**A, B**) and *Quercusserrata* (**C**) **D–F***T.dilaticeps* on Castanopsissieboldiisubsp.lutchuensis**G–I***T.robustus* on Castanopsissieboldiisubsp.sieboldii. Arrows indicate oviposition scars.

**Material examined.** Inohae-keikoku, Kitago, Nichinan, Miyazaki Pref., 16-VI-2019 (as larva on *Quercushondae*), emerged on 19-VII-2019 (Fig. 9A, B); Suizu, Tsuruga, Fukui Pref., 14-VII-2021 (as larva on *Q.glauca*), emerged on 21-VII-2021 (Fig. 9C); Suizu, Tsuruga, Fukui Pref., 19-VIII-2019 (as pupa on *Q.serrata*), emerged on 23-VIII-2019.

**13. *Trachysdilaticeps* Gebhardt, 1929**

Fig. 9D–F

**Host plant**. Fagaceae: Castanopsissieboldiisubsp.lutchuensis (Ohmomo and Fukutomi 2013).

**Leaf mine.** Gray full-depth linear-blotch mine on mature leaf. Egg is laid along midrib near leaf tip, and the mine expands downwards along leaf margin. Frass is granular and distributed all over the mine.

**Material examined.** Mt. Nishime, Kunigami, Okinawa Pref., 6-VI-2018 (as larva on Castanopsissieboldiisubsp.lutchuensis), emerged on 6-VII-2018 (Fig. 9D, E); Komi, Iriomote Is., Yaeyama, Okinawa Pref., 4-VI-2018 (as vacant mine of C.sieboldiisubsp.lutchuensis); Yakukachi, Sumiyo, Amami, Kagoshima Pref., 25-V-2017 (as larva on Castanopsissieboldiisubsp.lutchuensis), emerged on 15-VI-2017 (Fig. 9F).

**14. *Trachysrobustus* E. Saunders, 1873**

Fig. 9G–I

**Host plant**. Fagaceae: Castanopsissieboldiisubsp.sieboldii (Yano 1952).

**Leaf mine.** Gray full-depth linear-blotch mine on mature leaf. Egg is laid along midrib near leaf tip, and the mine expands downwards along leaf margin. Frass is granular and distributed all over the mine.

**Material examined.** Mt. Shizuhata, Aoi-ku, Shizuoka, Shizuoka Pref., 27-VI-2018 (as larva on Castanopsissieboldiisubsp.sieboldii), emerged on 13-VII-2018 (Fig. 9G, H); Suizu, Tsuruga, Fukui Pref., 11-VIII-2018 (as larva on C.sieboldiisubsp.sieboldii), emerged on 5-X-2018 (Fig. 9I).

**15. *Trachysminutussalicis* (Lewis, 1893)**

Fig. 10A–C

**Host plant**. Salicaceae: *Salixcaprea*, S.miyabeanasubsp.gymnolepis, *S.vulpina*, *Populusmaximowiczii* (Yano 1952), *Salixreinii* (new record).

**Leaf mine.** Brown full-depth blotch mine on mature leaf. Egg is laid just near leaf tip, and the mine expands downwards along leaf margin. Frass is granular and connected.

**Figure 10.**
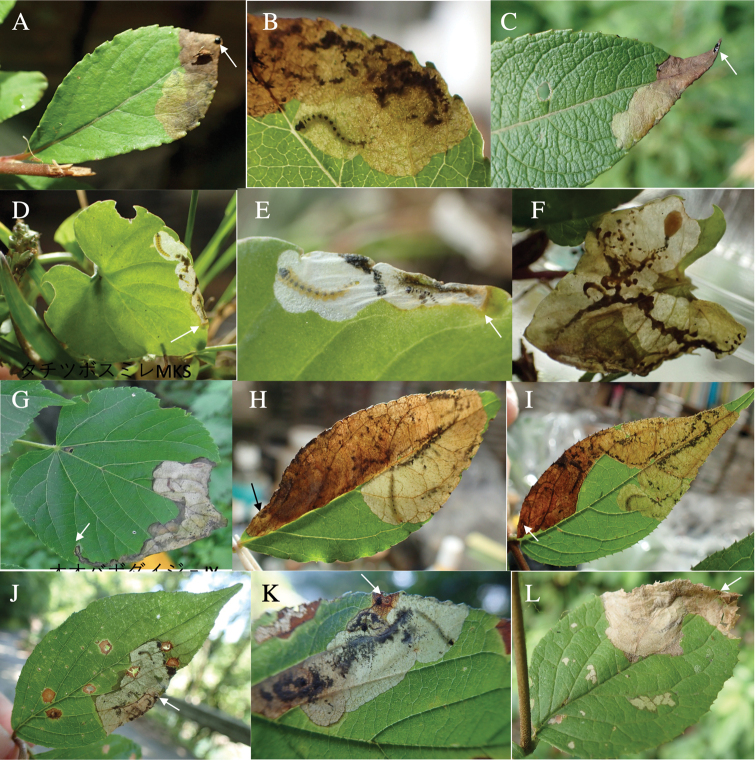
Leaf mines of *Trachys* spp. on leaves of Salicaceae, Violaceae, Malvaceae, and Hydrangeaceae. **A–C***T.minutus* on *Salixreinii* (**A, B**) and *Salixvulpina* (**C**) **D–F***T.pseudoscrobiculatus* on *Violagrypoceras***G***T.aurifluus* on *Tiliamaximowicziana***H, I***T.saundersi* on *Deutziacrenata* (**H**) and *Deutziagracilis* (**I**) **J–L***T.tsusimae* on *Deutziascabra*. Arrows indicate oviposition scars.

**Material examined.** Matsuo-kozan, Hachimantai, Iwate Pref., 7-VII-2018 (as larva on *Salixreinii*), emerged on 30-VII-2018 (Fig. 10A, B); Toyohara, Nasu, Nasu-gun, Tochigi Pref., 7-VII-2022 (as larva on *Salixcaprea*) (Fig. 10C).

**16. *Trachyspseudoscrobiculatus* Obenberger, 1940**

Fig. 10D–F

**Host plant**. Violaceae: *Violagrypoceras*.

**Leaf mine.** White full-depth linear-blotch mine on mature leaf. Egg is laid along leaf margin near leaf tip, and the mine expands downwards along leaf margin. Frass is granular and connected.

**Material examined.** Mt. Mikusa, Kato-shi, Hyogo Pref., 20-V-2018 (as larva on *Violagrypoceras*), emerged on 8-VI-2018 (Fig. 10D–F).

**17. *Trachysaurifluus* Solsky, 1875**

Fig. 10G

**Host plant**. Malvaceae: *Tiliamaximowicziana*, *T.japonica* (Kurosawa 1959).

**Leaf mine.** Brown full-depth linear-blotch mine on mature leaf. Egg is laid near leaf margin, and the mine expands along leaf margin. Frass is granular and distributed all over the mine.

**Material examined.** Iyari, Inao, Omachi, Nagano Pref., 1-VII-2013 (vacant mine of *Tiliamaximowicziana*) (Fig. 10G).

**18. *Trachyssaundersi* Lewis, 1893**

Fig. 10H, I

**Host plant**. Hydrangeaceae: *Deutziacrenata* (Yano 1952), *D.gracilis* (new record).

**Leaf mine.** Brown full-depth blotch mine of whole layers of leaf blade. Egg is laid along leaf margin near leaf base, and the mine expands upwards along leaf margin. Frass is granular and loosely connected.

**Material examined.** Makido, Niimi, Okayama Pref., 1-VII-2018 (as larva on *Deutziacrenata*), emerged on 17-VII-2018 (Fig. 10H); Donden, Sado, Niigata Pref., 13-VII-2019 (as larva on *Deutziacrenata*), emerged on 16-VIII-2019; Mt. Toyoguchi, Ooshika, Shimoina, Nagano Pref., 2-VIII-2020 (as larva on *Deutziagracilis*), emerged on 5-IX-2020 (Fig. 10I).

**19. *Trachystsusimae* Obenberger, 1922**

Fig. 10J–L

**Host plant**. Hydrangeaceae: *Deutziacrenata* (Ohmomo and Fukutomi 2013), but this record may be doubtful. *Deutziascabra* (new record). We obtained adults from only *D.scabra*.

**Leaf mine.** Brown full-depth blotch mine on mature leaf. Egg is laid along leaf margin, and the mine expands along leaf margin. Frass is granular and distributed all over the mine.

**Material examined.** Mt. Osuzu, Tsuno, Miyazaki Pref., 14-VII-2021 (as larva on *Deutziascabra*), emerged on 27-VII-2021 (Fig. 10J, K); Sakai-gawa, Takaoka, Miyazaki, Miyazaki Pref., 18-VII-2018 (as larva on *Deutziascabra*), emerged on 1-VIII-2018 (Fig. 10L).

**20. *Trachyscuneiferus* Y. Kurosawa, 1959**

**Host plant**. Unknown.

**Leaf mine.** Unknown.

## ﻿Discussion

After adding the two new species, we counted 34 trachyine species in the Japanese Archipelago (Table 1). Among these 34 species, host plants are known for 32, which are narrowly host-specific. All host plants are angiosperms belonging to ten orders of eudicots (Rosales, Fabales, Fagales, Malpighiales, Malvales, Cornales, Oxalidales, Myrtales, Santalales, and Ericales), suggesting that they are not associated with basal angiosperms, monocots, Saxifragales, Caryophyllales, or euasterids. From the standpoint of the life form of the host plant, 28 trachyine species are associated with woody plants (18 with trees, 10 with shrubs), three with subwoody climbing plants, and one with herbaceous plants. These results suggest that trachyine species have evolved from wood-borers associated with eudicots.

**Table 1.** A list of Japanese species of the tribe Trachyini, with their host plant species.

Buprestids		Host plants				
Genus	Species	Subclass	Order	Family	Genera	Habit
*Habroloma*	*subbicorne*	Rosids	Rosales	Rosaceae	*Rubus*	evergreen/ deciduous shrub
	*atronitidum*			Rosaceae	*Rubus*	deciduous shrub
	*marginicolle*			Rosaceae	*Rubus*	evergreen shrub
	*asahinai*			Rosaceae	*Rubus*	evergreen shrub
	*lewisii*			Rosaceae	*Rosa*	deciduous shrub
	*griseonigrum*		Fagales	Fagaceae	*Quercus*	evergreen/ deciduous tree
	*yuasai*			Juglandaceae	*Platycarya*	deciduous tree
	*elaeocarpusi* n. sp		Oxalidales*	Elaeocarpaceae*	Elaeocarpus*	evergreen tree
	*bifrons*		Geraniales?	Geraniaceae ?	*Geranium*?	perennial ?
	*nixilla insuicola*		Myrtales	Lythraceae	*Lagerstroemia*	deciduous tree
	*taxillusi* n. sp		Santalales*	Loranthaceae*	Taxillus*	evergreen epiphyte
	*eximiumeximium*	Asterids	Ericales	Symplocaceae	*Symplocos*	evergreen tree
	*eximiumeupoetum*			Symplocaceae	*Symplocos*	evergreen tree
	*liukiuense*			Symplocaceae	*Symplocos*	evergreen shrub
	*hikosanense*			?	?	?
*Trachys*	*auricollis*	Rosids	Fabales	Fabaceae	*Pueraria*	deciduous liana
	*reitteri*			Fabaceae	*Amphicarpaea*, *Glycine*, *Pueralia*, *Rhynchosia*	deciduous liana
	*tokyoensis*			Fabaceae	*Desmodium*	perennial
	*toringoi*		Rosales	Rosaceae	*Amelanchier*, *Chaenomeles*, *Cydonia*, *Malus*, *Pyrus*	deciduous tree
	*inconspicuus*			Rosaceae	*Prunus*	deciduous tree
	*pecirkai*			Ulmaceae	*Ulmus*	deciduous tree
	*cupricolor*			Ulmaceae	*Zelkova*	deciduous tree
	*yanoi*			Ulmaceae	*Zelkova*	deciduous tree
	*griseofasciatus*			Cannabaceae	*Aphananthe*, *Celtis*	deciduous tree
	*ineditus*			Cannabaceae	*Aphananthe*	deciduous tree
	*broussonetiae*			Moraceae	*Broussonetia*, Fatoua*	deciduous tree/ annual
	*variolaris*		Fagales	Fagaceae	*Quercus*	evergreen tree
	*dilaticeps*			Fagaceae	*Castanopsis*	evergreen tree
	*robustus*			Fagaceae	*Castanopsis*	evergreen tree
	*minutussalicis*		Malpighiales	Salicaceae	*Salix*, *Poplus*	deciduous tree
	*pseudoscrobiculatus*			Violaceae	*Viola*	perennial
	*aurifluus*		Malvales	Malvaceae	*Tilia*	deciduous tree
	*saundersi*	Asterids	Cornales	Hydrangeaceae	*Deutzia*	deciduous shrub
	*tsusimae*			Hydrangeaceae	*Deutzia*	deciduous shrub
	*cuneiferus*		?	?	?	?

* new records.

The host plant genera of the two new trachyine species are *Elaeocarpus* and *Taxillus*, belonging to Elaeocarpaceae (Oxalidales) and Loranthaceae (Santalales), respectively, and representing the first records in Tracheini for both families and orders, while both plant families have been recorded as hosts for *Agrilus* (Jendek & Poláková, 2014). The record on *Taxillus* is also the first record of buprestids associated with epiphytic, plant-parasitic plants.

Our records of leaf mines suggest that those of trachyine species are generally full-depth blotch mines on mature leaves of woody or subwoody plants, except for one species (*Trachyspseudoscrobiculatus*) associated with *Viola*. These leaf mines contrast with upper-layer mines on young leaves formed by agromyzids, epidermal/mesophyll mines on young leaves formed by gracillariids, thin full-depth linear mines formed by Lyonetiidae, and full-depth linear-blotch mines on young leaves formed by Eriocraniidae.

## Supplementary Material

XML Treatment for Habroloma elaeocarpusi

XML Treatment for Habroloma taxillusi
