# ﻿Corrigendum: Kato M, Kawakita A (2023) Diversity and larval leaf-mining habits of Japanese jewel beetles of the tribe Tracheini (Coleoptera, Buprestidae). ZooKeys 1156: 133–158. doi: 10.3897/zookeys.1156.97768

**DOI:** 10.3897/zookeys.1158.104708

**Published:** 2023-04-21

**Authors:** Makoto Kato, Atsushi Kawakita

**Affiliations:** 1 Graduate School of Human and Environmental Studies, Kyoto University, Sakyo 606-8501, Kyoto, Japan Kyoto University Kyoto Japan; 2 The Botanical Gardens, Graduate School of Science, The University of Tokyo, Tokyo, 112-0001, Japan The University of Tokyo Tokyo Japan

We recently published the description of two new buprestid beetle species *Habrolomaelaeocarpusi* and *Habrolomataxillusi* (Kato & Kawakita, 2023). However, no holotype depository is indicated in the paper. This is mandatory after 1999 according to the International Code of Zoological Nomenclature, and the new species names would be nomina nuda and unavailable (ICZN 1999: Art. 16.4.2). In this corrigendum, we indicate the holotype depository and correct the code numbers of the type specimens, which were incorrect. We thank Dr. Yutaka Tamadera (Hokkaido University) for kindly pointing out this error.

## ﻿ Habroloma elaeocarpusi sp. nov.

Taxon classificationAnimaliaColeopteraBuprestidae

0207F809-4D51-5A9D-B83E-1F5CF106CDF3

### Material examined.

***Holotype***: Japan: ♂ (MK-BP-a327), Mt. Osuzu, Tsuno-cho, Miyazaki Pref. NSMT-I-C-200345.

***Paratypes***: Japan: 1♂ (MK-BP-a360), same data as holotype, NSMT-I-C-200346; 1♀ (MK-BP-k35), Isso, Yakushima-cho, Yaku Island, NSMT-I-C-200347.

### Type depository.

The holotype and the paratypes are deposited at the National Museum of Nature and Science, Tokyo (NSMT).

## ﻿ Habroloma taxillusi sp. nov.

Taxon classificationAnimaliaColeopteraBuprestidae

B0E9B51B-5077-5A8D-8572-E77C05F34C59

### Material examined.

***Holotype***: Japan: ♂ (MK-BP-k40), Yakukachi, Amami-shi, Kagoshima Pref., NSMT-I-C-200348.

***Paratype***: Japan: 1♀(MK-BP-k39), same data as holotype, NSMT-I-C-200349.

### Type depository.

The holotype and the paratype are deposited at the National Museum of Nature and Science, Tokyo (NSMT).

## Supplementary Material

XML Treatment for Habroloma elaeocarpusi

XML Treatment for Habroloma taxillusi
